# Incongruences between morphology and molecular phylogeny provide an insight into the diversification of the *Crocidura poensis* species complex

**DOI:** 10.1038/s41598-022-12615-5

**Published:** 2022-06-22

**Authors:** Inessa Voet, Christiane Denys, Marc Colyn, Aude Lalis, Adam Konečný, Arnaud Delapré, Violaine Nicolas, Raphaël Cornette

**Affiliations:** 1 Institut de Systématique, Evolution, Biodiversité (ISYEB), Muséum national d’Histoire naturelle, CNRS, Sorbonne Université, EPHE, Université des Antilles, CP51, 57 rue Cuvier, 75005 Paris, France; 2 ECOBIO UMR 6553, CNRS-Université de Rennes 1, Paimpont, France; 3 Department of Botany and Zoology, Faculty of Science, Masaryk University, Brno, Czech Republic

**Keywords:** Ecology, Evolution, Speciation

## Abstract

Untangling the factors of morphological evolution has long held a central role in the study of evolutionary biology. Extant speciose clades that have only recently diverged are ideal study subjects, as they allow the examination of rapid morphological variation in a phylogenetic context, providing insights into a clade’s evolution. Here, we focus on skull morphological variability in a widely distributed shrew species complex, the *Crocidura poensis* species complex. The relative effects of taxonomy, size, geography, climate and habitat on skull form were tested, as well as the presence of a phylogenetic signal. Taxonomy was the best predictor of skull size and shape, but surprisingly both size and shape exhibited no significant phylogenetic signal. This paper describes one of the few cases within a mammal clade where morphological evolution does not match the phylogeny. The second strongest predictor for shape variation was size, emphasizing that allometry can represent an easily accessed source of morphological variability within complexes of cryptic species. Taking into account species relatedness, habitat preferences, geographical distribution and differences in skull form, our results lean in favor of a parapatric speciation model within this complex of species, where divergence occurred along an ecological gradient, rather than a geographic barrier.

## Introduction

Understanding morphological evolution, and the underlying mechanisms generating the tremendous phenotypic diversity, is a central aim in evolutionary biology^1^. The main drivers of morphological evolution are often grouped in two categories: internal constraints related to the genetic background of an individual or species (phylogeny, development, allometry…), and external, environment-related factors (climate, habitat, competition, predation…)^2^. Adult morphology is the outcome of complex genetic pathways, leading to the development and growth of the individual^3^. As a result of these genetic pathways, overall morphology may reflect evolutionary descent, with the retention of ancestral features following phylogenetic relatedness. In accordance with these expectations, it has been shown that morphological dissimilarity and divergence time can be positively correlated, meaning that recently diverged species tend to look more alike than more distant relatives^4^. Along with these developmental constraints, shape variation often occurs simultaneously with changes in size, which is known as allometry^5^. These changes may be evolutionary (bigger versus smaller species), ontogenic (younger, smaller individuals of the same species versus older, larger ones) or static (for a single age group, smaller individuals versus larger ones)^6^. As such, age and size may therefore affect shape, independently or in interaction. In addition to these pathways, morphology is strongly linked to functional demands, which are in turn dependent of the environment. As a consequence, morphological variation has long been considered a strong indicator of ecological preferences, to the point where morphology has been used as a proxy for ecological function^7^. The influence of the environment may relate to abiotic climatic conditions, as well as biotic interactions with the habitat, predators, preys, or competitors. To complicate matters further, morphological evolution may apply to an anatomical structure as a whole, but may also vary in intensity from one structure (or group of structures) to the next, with various degrees of correlation in their responses. These effects are known as modularity and integration^8^. In summary, the various combinations of random walk and selective forces and their relative strengths will affect the degree to which morphology will reflect phylogenetic history and/or the environment. The fact that these combinations may be different across taxa, anatomical structures, space, and time is a clear witness to the complexity of the processes involved in morphological diversification. Consequently, morphological diversity varies strikingly across clades^9^. Some groups present high levels of morphological disparity, regardless of their diversification rates or species richness^10^, while other groups contain high numbers of morphologically conservative species, whether a result of selection acting on body plan efficiency, or constrained body plans favoring higher diversity^11^.

The genus *Crocidura* (Eulipotyphla, Soricidae) is such an example of a morphologically conservative clade. It is the most species-rich of all extant mammal genera. The exact number of species still remains unknown due to the large diversity, widespread distribution, conservative morphology and general lack of genetic data for the genus^12^. This is especially the case for the African members of the genus, for which many attempts of classification have been made over the years, gathering them in groups of morphologically similar species also known as “species complexes”^13^. When traditional systematic methods fail to distinguish between species, leaving their number and phylogenetic relationships unknown, they are often referred to as cryptic species, grouped under a single name or with taxonomically unreliable denominations. Within the African shrews, the *Crocidura poensis* species complex was such an example. Recent works clarified the validity and phylogenetic relationships of its species^14–16^, but some speculation remains regarding the way the clade diversified.

The *C. poensis* species complex is widespread across tropical Africa, ranging from Senegal to Ethiopia. Twelve species are currently recognized in the most recent taxonomic checklist^12^, while only 10 lineages have been identified by recent molecular works^15,16^ (the three species *C. poensis*, *C. batesi* and *C. nigrofusca* were not confirmed by species delimitation analyses based on molecular data, and do not form monophyletic lineages). The 10 lineages can be roughly divided into two groups: the allopatrically distributed Central-East African lineages (*C. poensis* (Fraser, 1843); *C. fingui* Ceriaco et al., 2015; *C. turba* Dollman, 1910 and *C. similiturba* Konečný et al., 2020), and the West-African ones (*C. foxi* Dollman, 1915; *C. wimmeri* Heim de Balsac & Aellen, 1958; *C. grandiceps* Hutterer, 1983; *C. longipes* Hutterer & Happold, 1983; *C. theresae* Heim de Balsac, 1968 and *C. buettikoferi* Jentink, 1888), sympatrically distributed. They can be found over a wide array of habitats, ranging from open grasslands such as fields, savannas and fallows, to densely forested areas such as primary rainforests^12^. Most species are habitat specialists, with the exception of *C. buettikoferi* which can be found in various habitat types. All the species in the complex are believed to be terrestrial and nocturnal, but their feeding preferences and breeding habits remain poorly understood^12^. The diversification of the complex occurred during the Pleistocene, over a period of high climatic instability^15^. It has been hypothesized that the alternation between phases of cold/dry and warmer/wetter climate generated landscape changes which perhaps resulted in speciation events within the complex. Both geographic isolation (allopatric speciation) and ecological gradients (parapatric speciation) have been put forward as potential causes for its diversification, aided by previous works and other taxa^17^. In the first scenario, it has been proposed that populations may have found themselves geographically isolated in relic forest patches over drier climate bouts during the course of the Pleistocene’s climatic instability, this scenario being commonly referred to as the forest refugia hypothesis. Under this model of allopatric speciation in similar habitats, drift is expected to be the main force acting on the genome and morphology, and morphological disparity is expected to increase proportionally with divergence time, reflecting phylogenetic history^18,19^. In the second scenario, known as the ecotone model, an environmental gradient may have led to the divergence of species through habitat preferences^20^. Under this ecological gradient model, speciation is expected to occur as a result of habitat heterogeneity, and morphological differences should therefore be higher between two divergent habitat specialists, regardless of their relatedness. By exploring the conservative morphology of the complex with an integrative approach, this paper aims to disentangle its complicated morphological history and lend support or refute the diversification hypotheses put forward in Nicolas et al.^15^.

Using mitochondrial lineage identification and a geometric morphometric approach, cranial form (size and shape^21^) was examined within the *C. poensis* complex, testing for morphological differences between lineages. The skull is home to many vital sensory and cognitive organs. As such, skull morphology is governed by a multitude of functional constraints related to survival, such as feeding, predator evasion and partner detection and selection^22^. As is the case for overall form, the variety, the strength of the constraints and the differences in the rates of morphological evolution are responsible for the wide range of skull shapes and sizes observed within vertebrates. Skull morphology is therefore a good tool for investigating the diversity across and within species^23^, and sorting between different lineage diversification scenarios^24^.

The aim of this study was two-fold. First, the morphological diversity of the complex was quantified in order to test the role of internal (phylogeny, allometry) versus external (environment-related) constraints on its morphological evolution. Then, the results were examined in a phylogeographic framework, in an attempt to corroborate the diversification scenarios within the *C. poensis* species complex (geographic isolation versus ecological gradients).

**Figure 1:**
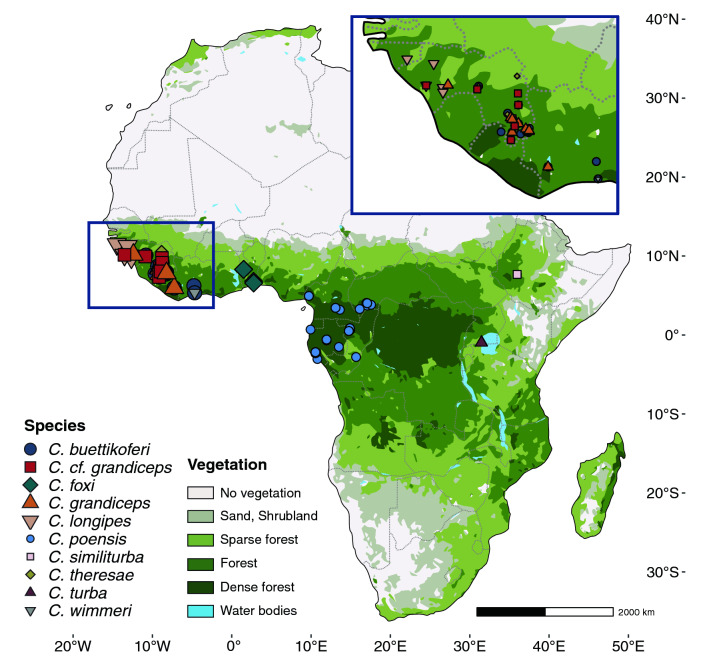
Specimen distribution map. Scaled map of Africa representing all specimens included in the morphometric study plotted over a schematic depiction of African biomes (vectorized rendition adapted from a google vegetation map, 2020, drawn with Procreate 5.2.6—https://procreate.art/—and vectorized using Adobe Illustrator 24.0.2—https://www.adobe.com/products/illustrator.html). Species are depicted using colors and symbols.

## Results

### Taxonomy: differences between species

**Figure 2:**
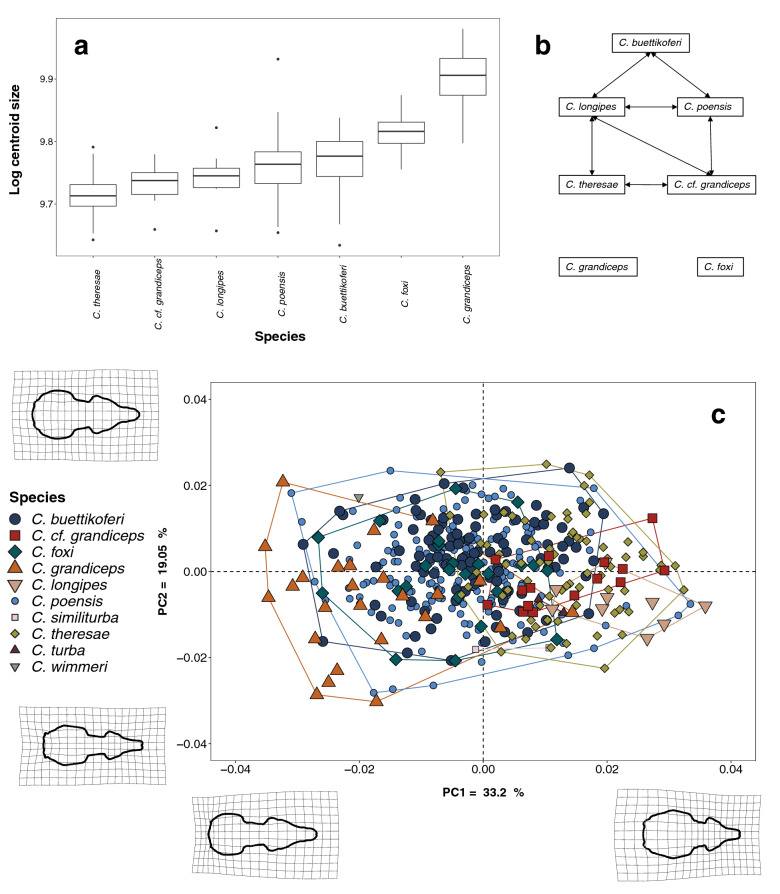
Size and shape variation within the C. poensis species complex. (**a**) Box plot with standardized deviation of log transformed centroid size for species for which N ≥ 9. (**b**) schematic depiction of size similarities. Arrows represent non-significant size differences. (**c**) Scatterplot of the two first PCs and their corresponding shape deformation grids relative to the mean shape, at the negative and positive extremes of the axes. All specimens are depicted, using colors and symbols for species, with minimum convex hull superimposed.

One-way ANOVA showed a significant effect of taxonomy on size in the whole dataset and the adults subsample (Mean sq = 0.13, F = 94.43 p ≪ 0.001 and Mean sq = 0.06, F = 52,15 p ≪ 0.001, Supplementary Table 2), and paired comparisons revealed size differences between *C. foxi* and *C. grandiceps* and all others (Supplementary Table 3; Fig. 2). The smaller species form a size gradient rather than discontinuous distributions. All extreme size values (biggest and smallest species) correspond to the highly sympatric westernmost African lineages, with the smallest species being *C. theresae*, and the largest *C. grandiceps* (Figs. 1, 2a,b). The PCA plot depicting the first two axes (Fig. 2c, PC1 = 33.2% of total variance, PC2 = 19.05% of total variance) shows high overlap between the different lineages, highlighting the difficulties regarding species identification. PC1 describes shape changes mostly visible in the rostrum to brain case ratio, negative values being associated with a rostrum elongation and broadening, an increased rostrum to brain case ratio, and a rounded brain case. PC2 describes changes in overall skull width and brain case roundness, with species at the extreme positive of the axes presenting broader skulls, and rounded brain cases. The MANOVA showed a significant effect of taxonomy on shape PCs in the whole dataset and the adults subsample (Table 1: R^2^ = 0.24, F = 22.22, p = 0.001 and R^2^ = 0.24, F = 11.89, p = 0.001 respectively), and paired comparisons showed significant shape differences between all species except for *C. cf. grandiceps* and *C. theresae* (Supplementary Table 4). Despite the interaction term between age and taxonomy in the full dataset for shape, results were comparable in the adult subsample.

**Table 1:** Multivariate regressions on skull shape for all specimens and adults only.

	Df	SS	MS	R^2^	F	Z	p value
**All specimens**							
Taxonomy	6.000	0.050	0.008	**0.241**	22.224	13.625	**0.001**
Age	4.000	0.018	0.005	**0.087**	10.188	8.899	**0.001**
Taxonomy × age	11.000	0.005	0.000	**0.026**	1.447	2.186	**0.013**
Sex	1.000	0.005	0.005	**0.023**	9.987	4.421	**0.001**
Taxonomy × sex	6.000	0.002	0.000	0.012	1.120	0.577	0.266
Size	1.000	0.027	0.027	**0.128**	63.389	8.590	**0.001**
Size × taxonomy	6.000	0.005	0.001	**0.024**	2.366	3.596	**0.001**
Habitat	7.000	0.017	0.002	**0.107**	5.896	7.307	**0.001**
Taxonomy × habitat	9.000	0.004	0.000	**0.028**	1.420	1.758	**0.041**
Latitude	1.000	0.007	0.007	**0.031**	14.011	5.022	**0.001**
Longitude	1.000	0.009	0.009	**0.042**	18.899	5.154	**0.001**
**Adults**							
Taxonomy	6.000	0.026	0.004	**0.243**	11.891	9.864	**0.001**
Sex	1.000	0.003	0.003	**0.031**	7.170	3.804	**0.001**
Taxonomy × sex	6.000	0.002	0.000	0.021	1.009	0.224	0.408
Size	1.000	0.016	0.016	**0.149**	40.545	6.374	**0.001**
Size × taxonomy	6.000	0.003	0.001	**0.032**	1.724	2.419	**0.008**
Habitat	6.000	0.010	0.002	**0.146**	4.478	6.132	**0.001**
Taxonomy × habitat	4.000	0.002	0.000	0.027	1.460	1.364	0.092
Latitude	1.000	0.004	0.004	**0.040**	9.697	4.500	**0.001**
Longitude	1.000	0.005	0.005	**0.044**	10.670	4.595	**0.001**

Significant values are in bold.

### Phylogenetic signal

**Figure 3:**
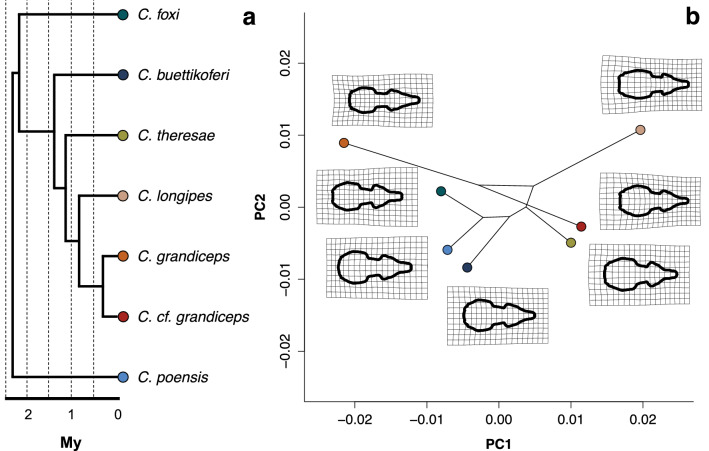
Species tree and phylomorphospace of the C. poensis species complex (only species with a specimen count ≥ 9 are shown). (**a**) Dated truncated species tree. (**b**) Phylomorphospace plot of PC1 and PC2 axes, for the same species. Values are based on species means. Deformation grids represent the mean for the species, relative to the mean for the whole data set, and amplified by a magnification factor of 3.

Both size and shape exhibited no significant phylogenetic signal (all specimens: K = 0.23, p > 0.9 & Kmult = 0.32, p > 0.9; adults: K = 0.23, p > 0.9 & Kmult = 0.33, p > 0.9), meaning the most closely related species do not look more alike than the more distantly related ones. The phylomorphospace representing only species for which N ≥ 9 displays this result. An apparent morphological convergence along axes 1 and 2 of the PCA for the species *C. theresae* and *C. cf. grandiceps* appears as well (Fig. 3)*,* and pairwise comparisons failed to detect any significant differences between these two species, in regards to size or shape (Supplementary Tables 3, 4).

### Allometry

**Figure 4:**
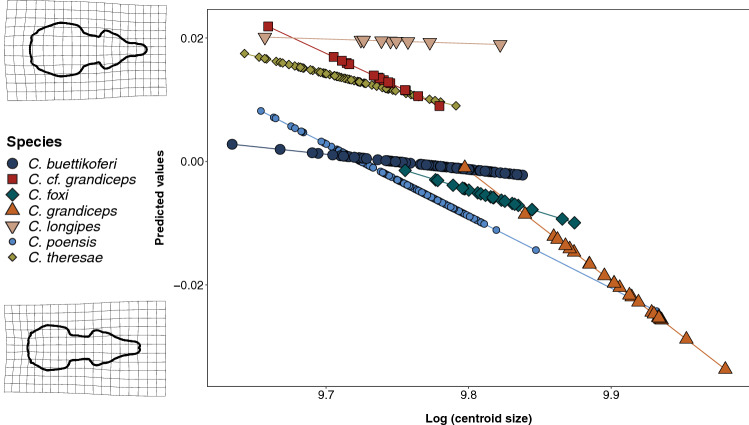
Allometric slopes of the C. poensis species complex (only species with a specimen count ≥ 9 are shown). Prediction lines representing predicted shape plotted against log centroid size with species grouping. Only species for which N ≥ 9 are shown. Deformation grids depict the shapes associated with the larger and smaller specimens, relative to the mean, with a magnification of 3. Species are labelled according to colors and symbols.

The MANCOVA used to test the effect of size on shape with a species grouping revealed an impact of size (Table 1: all specimens: R^2^ = 0.13, F = 63.39, p = 0.001; adults only: R^2^ = 0.15, F = 40.55, p = 0.001). After size was taken into account, taxonomy remained a significant predictor of shape. A weak yet significant interaction term existed between taxonomy and size (Table 1: all specimens: R^2^ = 0.02, F = 2.36 p = 0.001; adults: R^2^ = 0.03, F = 1.72, p = 0.008), indicating differences in the species-specific effects of size on shape. Pair comparisons were used to test the degree of the correlation between the allometric slopes of the various species, revealing three significantly different pairs (Fig. 4, Supplementary Table 5), *C. buettikoferi* and *C. poensis* (p = 0.002), *C. buettikoferi* and *C. grandiceps* (p = 0.04)*,* and *C. poensis* and *C. theresae* (p = 0.02). Pairwise differences in vector lengths showed five significant pairs: *C. buettikoferi* and *C. foxi* (p = 0.02), *C. buettikoferi* and *C. foxi* (p = 0.02), *C. buettikoferi* and *C. theresae* (p = 0.001), *C. foxi* and *C. longipes* (p = 0.04) and *C. poensis* and *C. theresae* (p = 0.002) (Supplementary Table 6). The effect of size on shape was significant in all species except for *C. longipes* and *C. cf. grandiceps* (Supplementary Table 7). R^2^ varied between 0.04 and 0.18, with the largest and smallest species having the largest allometric effect (R^2^ = 0.12 for *C. grandiceps* and *C. foxi,* the two largest, and R^2^ = 0.19 for *C. theresae*, the smallest; p = 0.02, 0.01 and 0.001 respectively). The two average-sized species, *C. poensis* and *C. buettikoferi*, had the lowest values of size effect (R^2^ = 0.06 and 0.04; p = 0.001 and 0.003 respectively).

### Environmental variables

All results from the statistical analyses can be found in Supplementary Table 2 for size and Table 1 for shape. The presence of a small yet significant geographical gradient was detected in skull shape (Latitude: R^2^ = 0.03, F = 14.01, p = 0.001; Longitude: R^2^ = 0.04, F = 18.90, p = 0.001), but not in skull size. However, the existence of a significant interaction term indicated this was likely due to the species distribution. The effect of habitat was significant both on size (R^2^ = 0.02, F = 8.97, p < 0.001) and shape (R^2^ = 0.10, F = 5.90, p = 0.001), but likewise, it could not be separated from taxonomy, due to the habitat exclusivity of most species. Overall, the smaller, more delicate-looking species seem to be present in open habitats such as savannas, grasslands and low-vegetation cultivated areas.

**Figure 5:**
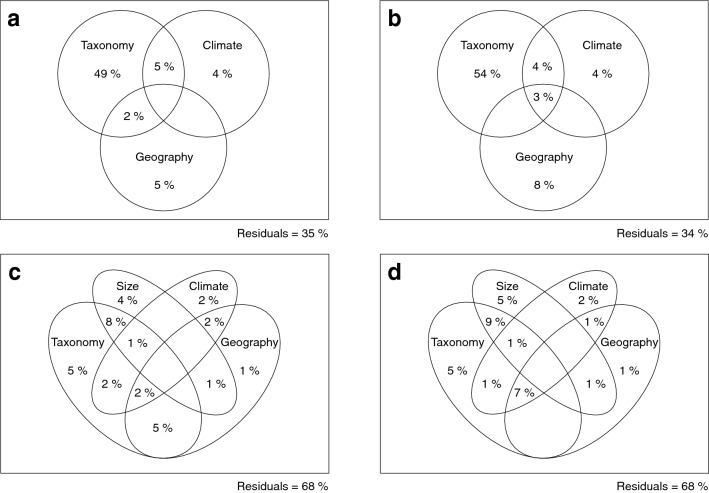
Variation partitioning in size and shape in the C. poensis species complex. Venn diagrams of the variation partitioning analyses for size in (**a**) all specimens, (**b**) adults, and shape in (**c**) all specimens and (**d**) adults. Percentages correspond to the total size and shape variation explained by circled factors (adj.R^2^). Values < 0 are not shown.

Variation partitioning analyses taking into account all relevant climate PCs as well as the geographic structure of the data retrieve the same results, with taxonomy and allometry explaining the bigger proportion of shape variation among the tested variables (5 and 4% respectively for all specimens, 5% each for adults; Fig. 5), taking into account that taxonomy is also highly significant factor of size differences. Climate remains a weak yet significant predictor (2% for all specimens and adults), and a large proportion of the variance remains unexplained (68% for both).

**Figure 6:**
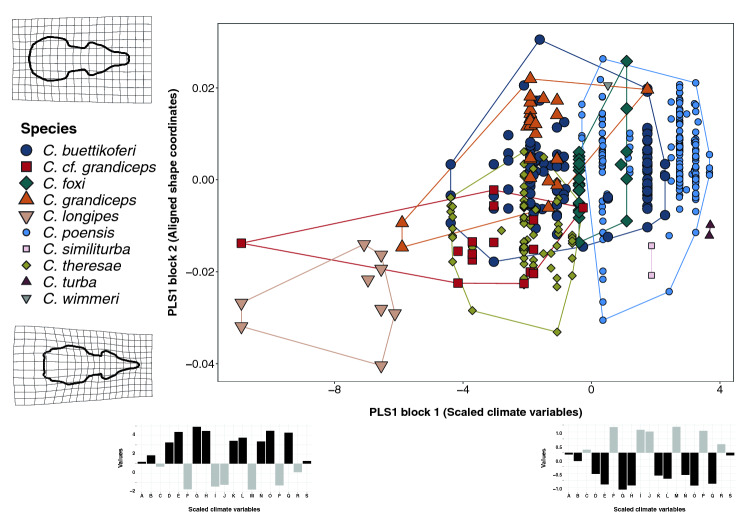
Two-block partial least square regression of shape and climate. Scatterplot of the first pair of Singular Warps of the Two Block Partial Least Squares analysis between scaled climatic variables (X axis) and symmetrical shape coordinates (Y axis). Deformation grids represent the shape for the minimum and maximum values of the first shape PLS axis plotted against the mean, with a magnification of 3. The climate variable plots represent the loadings of each original variable for the minimum and maximum values of the first climate PLS axis. The variables are as following, the bold ones being the main contributors: A: Annual mean temperature; B: Mean diurnal range; C: Isothermality; D: Temperature seasonality; E: Max temperature of warmest month; F: Min temperature of coldest month; G: Temperature annual range; H: Mean temperature of wettest quarter; I: Mean temperature of driest quarter; J: Mean temperature of warmest quarter; K: Mean temperature of coldest quarter; L: Annual precipitation; M: Precipitation of warmest month; N: Precipitation of driest month; O: Precipitation seasonality; P: Precipitation of wettest quarter; Q: Precipitation of driest quarter; R: Precipitation of warmest quarter; S: Precipitation of colder quarter.

The Two Block Partial Least Squares analysis between the climatic variables and the shape coordinates found a correlation coefficient of 0.48 (p = 0.001). The first 2 PLS axes explain 80% of the total covariance. The scatter plot (Fig. 6) shows a taxonomic gradient along the climatic gradient, where the larger number of individuals and species is located towards the null and positive values of the first PLS axis for the climate variables. These values are associated with lower temperature annual range and precipitation seasonality, an indicator of stabler climate. The two sympatric species with larger size and shape differences (the massive *C. grandiceps*, and the very small *C. theresae*) are present toward more negative values along the first PLS axis, associated with slightly higher climatic variability. The furthest left on the scatter plot is *C. longipes*, a species located in the westernmost part of tropical Africa. The species located in areas with the highest values of temperature annual range and precipitation seasonality are overall the smallest ones, exhibiting the associated delicate-looking skull anatomy, with the reduced, narrower rostrum, proportionally broader brain case, and accentuated paraoccipital processes.

## Discussion

The species of the *C. poensis* complex differ slightly regarding dorsal skull morphology. Taxonomy is the best predictor of dorsal skull size and shape, regardless of age. Both the subtlety of the morphological differences and the variance in skull shape and size are responsible for the substantial overlap, creating a morphological gradient rather than a clearly discriminating morphospace, thereby explaining the difficulties regarding species identification, as is common in speciose small mammal clades. Of the highly overlapping West African lineages, *C. grandiceps* is the only one that can be discriminated from the other species of the complex based on dorsal skull size and shape. Furthermore, both size and shape extremes are among the geographically overlapping West African species (*C. theresae, C. cf. grandiceps, C. grandiceps*). Under the competitive exclusion hypothesis, species that are ecologically too similar should not be able to co-occur^25^. As such, when two species show too many morphological or ecological similarities, a change in size, shape, function or any combination of the latter is expected in order to reduce competitive pressures. This form of divergent morphological evolution could explain the fact that both the largest and the smallest species, *C. theresae* and *C. grandiceps* (also representing the extremes of the morphospace) occupy roughly the same geographic area. It is also of note that these two species occupy different habitats, *C. theresae* being almost exclusively limited to open landscapes such as savannas, fields and fallows, and the larger *C. grandiceps* being an exclusive forest dweller^15,26^. Habitat preferences play a key role in facilitating the co-occurrence of morphologically similar species^27^. This usually entails a shift in ecological preferences along with a change in size and/or shape. Additionally, divergent selection on skull morphology is not sufficient to explain the patterns observed within the *C. poensis* species complex. The yet undescribed species *C. cf. grandiceps*, closest relative of the largest *C. grandiceps*, exhibits a size and shape undistinguishable from the smallest *C. theresae*. These three species occur sympatrically in Guinea, Liberia and Ivory Coast. Contrary to its exclusive forest-dwelling sister species, some *C. cf. grandiceps* specimens were captured in open landscapes^26^. This apparent morphological convergence between *C. cf. grandiceps* and *C. theresae* in potentially competing species (they occupy both the same geographical region and same habitat) is counterintuitive, but numerous other cases have been reported in the literature, where some small mammal species exhibit sympatric populations showing higher similarity among them than with their allopatric intraspecific representatives, whether these trends reflect local plasticity or long-term evolutionary trends^28^. Likewise, some clades may present very similar patterns of morphological evolution in spite of high levels of sympatry^29^.

As form is primarily governed by an individual’s genetic background, it is expected for morphological similarity to reflect a clade’s phylogeny^3^. This has been reported in multiple clades^30,31^, but was not the case is the *C. poensis* species complex, which is an exception rather than the rule. Other exceptions have been described^32,33^, and are justified by the fact that morphology can be affected by many different factors (see “Introduction” section), and that morphological convergences are much more common than amino acid ones^34^. Convergences, known as homoplasy, are misleading: the phenotypic similarities are confused with homologies, leading to inconsistencies between morphological and molecular phylogenies^35^. When morphology is quantified over a molecular phylogeny base, this translates as an absence of phylogenetic signal, that is, of the absence of a relationship between the degree of species relatedness and morphological similarity^36^. According to Caumul and Polly^30^, the difference between studies recording significant phylogenetic signal in morphometric data, and those failing to do so is partly due to the recency of common ancestry. However, the situation is not so simple. Perhaps due to the arbitrary nature of taxonomic levels, examples of the presence and absence of phylogenetic signal in morphological data can be found at multiple taxonomic levels, within a single genus, order^33^, or between many orders^37^.

The second strongest predictor for shape, after taxonomy, is size. In the global species pool, the smaller ones such as *C. theresae* and *C. cf. grandiceps* are characterized by a shorter rostrum, and a proportionally larger brain case with exaggerated paraoccipital processes. The largest species such as *C. foxi* and *C. grandiceps* exhibit a rounded braincase along with an elongated rostrum. This is common in mammals, where larger skulls often correlate with a lengthening of the face^38^. The effect of size on shape can be characterized by an allometric slope, differing in length and/or direction. The direction, or slope, corresponds to the strength of the allometric effects, which reflects the degree at which a variation in size might be associated with a shape change. The species of the complex do not exhibit the same responses to size increase. The allometric vectors are shorter in smaller species (regardless of sample size), yet the slope value is not higher in larger species only. Here, closely related species do not necessarily share similar slope values. The three pairs presenting different allometric slopes are not the most phylogenetically distantly related ones, which in this case does not support the hypothesis that allometric trajectories grow apart as divergence time increases^39^. When looking at individual species, size had a large effect on shape in the largest *C. grandiceps* and *C. foxi*, but also in *C. theresae,* and two of the highest slope values are among the recently diverged species *C. grandiceps* and *C. theresae*. Allometry can represent an easily accessed source of variability, as changes in size partner with shape differences, allowing slight variations in function^40^. Variations in rostrum length, and overall skull and maxillary width have been associated with shifts in bite force, and increases in prey size and hardness range^41^. In the *C. poensis* complex, the last common ancestor of the West African lineages probably occurred in the Guinean Rain forest^15^, and the forest dwellers in our dataset show in average larger skull sizes than the open landscape inhabitants. Small body size may have been selected in certain lineages or populations as it may increase fitness by facilitating predator evasion, regardless of the environment^42^. Small body size may also arise as a consequence of population density in certain areas^43^. Considering the relatively strong effect of size on shape for certain species, and the knowledge that allometry can serve as an easily accessible source of variation, it may have represented a very useful tool in the rapid diversification of the group, under stressful situations of competition for food resources and predator evasion.

No overall geographic gradients could be detected in skull size, and only very small latitudinal and longitudinal trends were recovered in skull shape. However, those could not be separated from the effect of taxonomy. The relative stability of temperatures along the Equator^44^ could explain the absence of a latitudinal size gradient, yet considering the phylogeography of the *C. poensis* complex, a stronger longitudinal gradient might have been expected. Its relative absence is likely due to the higher disparities found among the sympatric West African species, and the lack of phylogenetic signal in the morphological data. In the variation partitioning analyses, the combined climatic variables explain a similarly small portion of size and shape variation, and the two block PLS displays once more a taxonomical gradient in shape. The current effect of geography and climate may not be strong, but climate in the tropics is very stable nowadays, which has not always been the case. Although global trends are not marked in morphology today, it does not exclude past conditions from having impacted the diversification of the group.

The weak influence of global geography and current climate, the dominating effect of taxonomy on skull size and shape, in addition to the absence of a phylogenetic signal in either size or shape seem to indicate that past local selective pressures probably forged the subtle morphological diversity observed today. In the case of the *C. poensis* species complex, morphological differentiation may be strongly correlated with speciation times. All diversification events of the complex took place during the Pleistocene^15^, a period of high climatic instability^44^. The alternating climatic phases led to dramatic changes in the landscape, with gradual forest expansions and retractions, leaving patches of open, drier habitats. Of the two hypotheses proposed for the diversification of the West African lineages, one appears more likely. In the refugia hypothesis, populations are isolated through habitat discontinuity during unsuitable climate periods, and diverge through time mainly under genetic drift^18^. In this case, populations remain in the same habitat, and niche conservatism is expected^19^. Morphological divergence is expected to carry a phylogenetic signal, that is, to evolve according to a Brownian Motion, which was not the case here, at least not in West-African lineages. In the second scenario, the ecotone model, species diverge along an ecological gradient according to habitat preferences^20^. In this case, as diversification occurs along a landscape gradient, morphology is expected to respond to habitat-specific selective pressures. Thus marked morphological differences are awaited between sister species having diverged according to this model, and no phylogenetic signal in the morphological data is expected^45^. However, reality is not always so simple and ecological differences can also accumulate after an allopatric speciation. As climate shifts occurred, refugia expanded and genetically differentiated populations may have come in secondary contact, resulting in competition between the ecologically similar species and leading to character displacement^46^. Here, the five broadly sympatric species in West Africa do not have exactly the same habitat requirements^15^: *C. buettikoferi* can be found in a wide range of habitats such as grassland within the rain forest, forests, forest relicts in derived savanna, fallows and cocoa plantations; *C. grandiceps* is considered a forest species, *C. theresae* is mostly captured in open habitats such as grasslands and fields and only occasionally in forest relicts; *C. longipes* is only found in Western Coastal Guinea in small gallery forests isolated in savannas; *C. cf grandiceps* is found in forests while some individuals were captured in open landscapes. Important size, with no overlap, and shape differences were found between *C. grandiceps* and the four other species. On the morphospace (Fig. 3), all these species appear well differentiated, except *C. cf grandiceps* and *C. theresae*. These last two are not sister species in the phylogenetic tree and they seem to occasionally cohabit in similar habitats. These results, along with the fact that no phylogenetic signal could be detected, tend to support the ecotone model. However, it is important to note a large overlap is observed on the PC1-2 plane, suggesting that morphological differences remain rather subtle. Despite the fact that the differences in skull width and rostrum length captured on the dorsal view allow to distinguish between certain species of the complex, other structures present on the more commonly used ventral face might provide better discrimination tools. Finding a lack of phylogenetic signal on the landmarks of the ventral face would corroborate the fact that the morphological evolution of the skull of shrews belonging to the C. poensis complex was not an entirely neutral process. Lasty, collecting more individuals from the Eastern species would provide an interesting comparison with the sympatric Western species, as most of the Eastern ones occur allopatrically, and do not compete with one another.

## Materials and methods

### Specimens and species identification

Following Nicolas et al.^15^, and in order to assign each specimen to a species, we sequenced the Cytochrome b (Cytb) and/or 16s rRNA (16S) mitochondrial gene of all specimens included in our morphometric analyses. The biological materials come from the Muséum national d’Histoire naturelle (Paris, France) and the Národní muzeum (Prague, Czech Republic). NJ trees were reconstructed in Geneious prime version 2021.1.1 (https://www.geneious.com), both for the Cytb and 16S genes and a mitochondrial lineage was assigned to each specimen. *C. fingui*, endemic to Príncipe Island^14^, was excluded from our analyses as no intact skulls could be retrieved. Due to several very small sample sizes, three additional species could not be included in the analyses taking into account taxonomy: the East African species *C. turba* and *C. similiturba*, and the critically endangered *C. wimmeri* endemic to Ivory Coast^47^. Specimens originally described as *C. grandiceps* were divided into two lineages, *C. grandiceps* and a monophyletic group of smaller individuals temporarily named *C. cf. grandiceps* while awaiting formal description. Details regarding this choice may be found in SM1 (methods section). The analyses including taxonomy as a factor focused on the 7 remaining species (species with N ≥ 9), 6 of which are distributed in West Africa. In total, 4 datasets were generated: (1) all specimens of all species (N_1_ = 433), (2) adults of all species (N_2_ = 233), (3) all specimens of species for which N ≥ 9 (N_3_ = 428) and (4) adults of species for which N ≥ 9 (N_4_ = 221). The choice of examined lineages and their individual count per lineage can be found in SM1 (methods section and Table 1), and the specific individuals included in each subset can be found in SM2.

### Data acquisition

A recent study focusing on *Crocidura* species from Sulawesi showed that skull size, rostral length and skull width are useful criteria for species discrimination^48^. All three are captured in the complete outline of the skull in dorsal view, using skull centroid size as a proxy for overall size. Due to the small size, the fragile nature, and the high likeliness of *Crocidura* specimens, using the outlines allowed the inclusion of more individuals while capturing the variability in overall skull shape. In addition, the use of an automated procedure avoided the biases caused by manual landmark placement^49^. 433 genotyped individuals of the *C. poensis* species complex were photographed using a NIKON D5600 and a 60 mm AF-S Micro NIKKOR lens. The individuals and their information are displayed in SM2. Specimens were assigned an age group using a combination between suture fuse of the basioccipital and basisphenoid bones and tooth wear (Supplementary Fig. 2), to verify the possible interaction between species and age effects on shape. The outline curves and a single homologous landmark were used in the morphometrics protocol detailed in SM1, outputting symmetrical Procrustes coordinates, centroid size (which was log transformed), and PC axes retaining 90% of total shape variability.

### Phylogenetic signal

One representative specimen for each species for which N ≥ 9 was used to generate a dated species-level tree. Details on the methodology can be found in SM1. Using the species tree, the presence of a phylogenetic signal was tested in the corresponding species’ skull size and shape. The univariate K-statistic and its multivariate equivalent Kmult^50,51^ were calculated on average shape coordinates and mean centroid size for each species, using *phylosignal* & *physignal* from the *picante*^52^ *& geomorph* packages. These approaches compare the value of a trait against the theoretical value of that trait evolving under a Brownian motion model, using a provided phylogeny, and test whether closely related species resemble each other more than more distantly related ones. Using the shape PCs, the species were plotted in a phylomorphospace with the *phylomorphospace* function of the *phytools* package (Fig. 4, Ref.^53^).

### Environmental variables

Due to the wide geographic distribution of the complex, the existence of a latitudinal, longitudinal, and climatic gradient was investigated in the skull morphology of the global species pool. The 19 climatic variables were extracted from the GPS coordinates using the WordClim2 raster database (https://www.worldclim.org/, Ref.^54^) with a 2.5 arc-minute resolution and the *st_within* function from the *sf* package^55^. Since many of the climatic variables are highly correlated, a PCA was run separately on the datasets of numerous specimens and numerous adults subsample (N_3_ and N_4_), using *prcomp* in the *stats* package, to extract synthetic uncorrelated climatic variables. In order to account for spatial autocorrelation caused by the geographic distribution of our samples, principal coordinates of neighbor matrices (PCNMs) were calculated with the *vegan* package^56^ using the truncated geographic distance matrix^57,58^. These PCNMs correspond to spatial filters representing geographic distance. A forward selection was performed to select only the climate PCs and spatial filters significantly correlated with shape and size, using *forward.sel* from the *adespatial* package^59^. Collection habitat was simplified into broad categories depending on vegetation density. All habitats and their simplifications can be found in SM2.

### Allometry and age effect

The effect of taxonomy and age were first tested, both on size and on shape using ANOVA and MANOVA (*aov,* from *stats*; *procD.lm,* from *geomorph*). Due to the existence of a significant interaction term between taxonomy and age on shape, all subsequent analyses were run on the entire dataset as well as on adult specimens separately, to verify that any detected effect would remain significant regardless of age. In addition, even though size and shape are extracted as separate components from the GPA, the shape component still contains some size information^6^. Size-free shape coordinates (residuals of a multivariate regression of shape on size) could not be retained due to an interaction term between taxonomy and size, witness to the absence of a common allometric slope between all species, and size was kept as a covariate^6^. After size was regressed against shape with a species grouping, the allometric slopes of the different species were compared in the numerous specimens dataset (N_3_; *pairwise* function, *RRPP* package^60^). The significance of size on shape was tested to estimate the strength of allometry in each individual species from N_3_, by subsetting the raw outlines and running the morphometrics protocol on each species data set separately.

### Pooled species samples

The absence of a significant interaction term between taxonomy and sex both for size and shape (p > 0.05) justified sex pooling. The effects of taxonomy, habitat, latitude, and longitude were tested separately on size and on shape, to avoid confusion from multiple interaction terms. As mentioned above, the effects were tested both on the whole data set and the adult subsamples (N_1_ through N_4_ depending on whether taxonomy was considered as a predictor), to control for age effect (size: Supplementary Table 2 & shape: Table 1). Pairwise differences between species were tested on the datasets of numerous specimens and numerous adults subsample (N_3_ and N_4_). Significant size differences were examined using a post-hoc t-test with Bonferroni correction to account for multiple testing (*pairwise_t_test from the rstatix* package^61^; Supplementary Table 3). Forms were compared two by two to test for statistically significant discrimination between species using the *pairwise* function (Supplementary Table 4). The relative contribution of taxonomy, climate (all relevant PCs) and geography (represented by the relevant spatial filters) was calculated using variation partitioning (Ref.^62^; *varpart* in the *vegan* package) both for the whole dataset of numerous specimens and the numerous adults subsample (N_3_ and N_4_). The results are displayed with a schematic Venn Diagram representing the percentage of variation explained by each of the examined factors (Fig. 5). In order to calculate the covariance between shape and climate, and visualize the climatic gradient in skull shape, a two-block partial least square regression^63^ was run on shape data and scaled climatic variables for the complete dataset using a combination of *tw2.b.pls* from *geomorph* and *pls2B* from the *Morpho* package^64^ (Fig. 6).

## Supplementary Information

Supplementary Information 1.

Supplementary Information 2.

## Data Availability

Cytb and 16S sequences have been uploaded to Genbank, and accession numbers can be found in the specimen table (SM2). A TPS file containing all specimens and their sliding semi-landmarks has been uploaded to https://data.indores.fr/.
