# Supplementary material for: Incongruences between morphology and molecular phylogeny provide an insight into the diversification of the Crocidura poensis species complex
Source: Sci Rep. 2022 Jun 22;12:10531. doi: 10.1038/s41598-022-12615-5 (PMC9217945; doi:10.1038/s41598-022-12615-5)
Supplement: Supplementary file 1 — Supplementary Information 1. [file 41598_2022_12615_MOESM1_ESM.docx]

Incongruences between morphology and molecular phylogeny provide an insight into the diversification of the *Crocidura poensis* species complex

Inessa Voet^1^*, Christiane Denys^1^, Marc Colyn^2^, Aude Lalis^1^, Adam Konečný^3^, Arnaud Delapré^1^, Violaine Nicolas^1a^*, Raphaël Cornette^1a^*

^a^ co-last authors

* Corresponding authors

Inessa Voet [inessa.voet@mnhn.fr](mailto:inessa.voet@mnhn.fr)

Violaine Nicolas [violaine.colin@mnhn.fr](mailto:violaine.colin@mnhn.fr)

Raphaël Cornette [raphael.cornette@mnhn.fr](mailto:raphael.cornette@mnhn.fr)

1 Institut de Systématique, Evolution, Biodiversité (ISYEB), Muséum national d'Histoire naturelle, CNRS, Sorbonne Université, EPHE, Université des Antilles, CP51, 57 rue Cuvier, 75005 Paris, France

2 ECOBIO UMR 6553, CNRS-Université de Rennes 1, Paimpont, France

3 Department of Botany and Zoology, Faculty of Science, Masaryk University, Brno, Czech Republic

# Supplementary Material 1: Methods and results

**Data acquisition.** Pictures were taken on a macrophotography stand, after adjusting camera angle with a spirit level and by a single individual to avoid inter-observer error. They were batch cropped and binarized using ImageJ version 1.53a [1]. When possible, skulls had been cleaned manually before taking the pictures, by removing remaining dry flesh interfering with the silhouette. In cases where the flesh was too dry and impossible to remove, the binarized pictures were cleaned manually by smoothing out the projected flesh. Binarized images were imported into R version 4.0.5 [2] and the outline was extracted, smoothed, centered and rotated using the *import_jpg*, *coo_smooth,* *coo_center* and *coo_rotate* functions in the *Momocs* package [3]. Skulls were not scaled at this point, to allow the later retrieval of centroid size. Centering and rotating were performed to enable the automatic placement of a single homologous landmark at the back of the skull, using the intersection between the skull’s back curve and the null intercept on the centered, rotated outlines. 300 sliding semi-landmarks [4] were sampled along the curve, and a Generalized Procrustes Analysis (GPA, [5]) was performed using *gpagen* in the *geomorph* package [6], allowing the sliding semi-landmarks to glide along the average curve while minimizing bending energy [7]. The use of many sliding semi-landmarks allows the retention of most of the information on the outline, without limiting the possibilities in subsequent analyses compared to the use of landmarks. The GPA removes differences due to size, orientation and position, and by allowing the sliding semi-semilandmarks to slide, they converge to similar positions on the outline and can then be used in the same way as homologous landmarks in the subsequent analyses [7]. This procedure outputs morphological data in two forms: aligned Procrustes shape coordinates, and centroid size, which was log transformed. The function *bilat.symmetry* was then used on the shape coordinates and only the symmetrical component was kept, removing measure or stress related asymmetry in the skull [8]. Principal Component Analyses (PCA) were run on the symmetrical component of aligned shape coordinates. The axes retaining 90% of total shape variability were used in a series of multivariate regressions [9]. To evaluate the validity and reproducibility of this method, a repeatability test was performed on 3 specimens belonging to the same species (*C. poensis*), sex, locality, habitat and age group. The series of 3 was photographed 11 times, repositioning the camera and skull between each take, and the morphometric method described above was used on the whole set. The PCA showed three distinct groups with very little intra-individual variability compared to the inter-individual variability (SM1 figure 1).

**Species tree.** Ten lineages were initially taken into account; 9 had been identified using species delimitation approaches (*C. poensis*, *C. fingui*, *C. turba*, *C. buettikoferi*, *C. theresae*, *C. longipes*, *C. grandiceps*, *C. wimmeri*, and *C. foxi*) [10] and a tenth, *C. similiturba,* was recently described based on genetic and morphometric data [11]. After running the analyses using these 10 taxonomical units, *C. grandiceps* displayed twice as much morphological variability as the other species, with two distinct clusters at opposite sides of the morphospace. Molecular phylogenetic analyses for Cytb sequences recovered two well supported monophyletic clades partitioning the large and smaller *C. grandiceps* individuals (unpub. Data). These results suggest that we do not have one, but two cryptic species. Comparison with the type specimen of *C. grandiceps* suggest that the smaller specimens are a part of a new species that we renamed *C. cf. grandiceps* while awaiting formal description (in progress). Divergence times for these 11 lineages were estimated from the combined supermatrix of mitochondrial (Cytb, 16S and CO1) and nuclear (BRCA1, STAT5, HDAC2) sequences. Partition Finder 2 [12] was used to select a subset scheme and substitution models as assessed by the BIC. Input data blocks were defined by gene and codon positions and settings specified exploration of all models available in Beast and all subset schemes given the data blocks. The best-fit scheme proposed seven subsets: (BRCA1-pos1, BRCA1-pos2, BRCA-pos3, HDAC2) (STAT5) (16S) (Cytb-pos1) (CO1-pos3, Cytb-pos2) (Cytb-pos3, CO1-pos1) and (Co1-pos2). Partitions and models were set according to Partition Finder 2 results. BEAST V1.8.4 [13] was used, assuming a Yule model of speciation and an uncorrelated log-normal distribution molecular clock as tree priors. The prior for ucld.mean was defined by continuous-time Markov chain rate reference, as advised when no prior knowledge is available [14]. Clock and sites models were unlinked across partitions. The MCMC simulations were run twice with 60 million iterations, with genealogies and model parameters sampled every 6000 iterations. The program TRACER [15] was used to assess algorithm convergence. To ensure chain stabilization, a burn-in representing the first 25% of trees was removed. LogCombiner v1.8.4 [16] was used to combine results from the two runs and TreeAnnotator v1.8.4 [16], to compute the maximum clade credibility tree. Fig-Tree 1.4.2 (http://tree.bio.ed.ac.uk/software/figtree/) was used to visualize the maximum clade credibility tree and the associated chronogram. Three secondary calibration points derived from the study of Dubey et al. [17] were used to calibrate the phylogeny, following the methodology described in Nicolas et al. [10].

#

Figure 1: Scatterplot of the two first PCs from the PCA run on three individuals from the C. poensis species (same locality, same sex, same age).

Figure 2: Age class determination chart, by degree of tooth wear and suture fuse.

Table 1: Specimen count for the whole data set and adults only. Only species for which N ≥ 9 were included in the analyses taking into account taxonomy (in bold).

| **Species** | **All age groups** | **Adults only** |
| --- | --- | --- |
| ***C. buettikoferi*** | **107** | 65 |
| ***C. cf. grandiceps*** | **15** | 8 |
| ***C. foxi*** | **24** | 14 |
| ***C. grandiceps*** | **25** | 10 |
| ***C. longipes*** | **9** | 9 |
| ***C. poensis*** | **174** | 75 |
| *C. similiturba* | 2 | 2 |
| ***C. theresae*** | **74** | 48 |
| *C. turba* | 2 | 2 |
| *C. wimmeri* | 1 | 0 |

Table 2: Univariate regressions of dependent variables on size for all specimens and adults only. Thick lines separate the regressions as they were run. Significant results are shown in bold.

|  | **Df** | **Sum Sq** | **Mean Sq** | **F value** | **Pr(>F)** |
| --- | --- | --- | --- | --- | --- |
| ***All specimens*** |  |  |  |  |  |
| **Taxonomy** | 6 | 0.75 | **0.13** | 94.43 | **0** |
| Age | 4 | 0.02 | 0.01 | 1.92 | 0.106 |
| Taxonomy * Age | 11 | 0.02 | 0.00 | 1.48 | 0.136 |
| **Sex** | 3 | 0.09 | **0.03** | 9.93 | **0** |
| Taxonomy * Sex | 7 | 0.01 | 0.00 | 0.62 | 0.739 |
| **Habitat** | 7 | 0.17 | **0.02** | 8.97 | **0** |
| Taxonomy * Habitat | 9 | 0.01 | 0.00 | 0.62 | 0.784 |
| Latitude | 1 | 0.00 | 0.00 | 1.30 | 0.255 |
| Longitude | 1 | 0.00 | 0.00 | 0.67 | 0.415 |
| ***Aldults*** |  |  |  |  |  |
| **Taxonomy** | 6 | 0.35 | **0.06** | 52.15 | **0** |
| **Sex** | 3 | 0.06 | **0.02** | 8.06 | **0** |
| Taxonomy * Sex | 6 | 0.00 | 0.00 | 0.41 | 0.872 |
| **Habitat** | 6 | 0.09 | **0.01** | 7.40 | **0** |
| **Taxonomy * Habitat** | 4 | 0.01 | **0.00** | 3.35 | **0.012** |
| Latitude | 1 | 0.00 | 0.00 | 0.36 | 0.552 |
| Longitude | 1 | 0.00 | 0.00 | 0.00 | 0.962 |

Table 3: Size comparisons between species for all specimens and adults only. Values were obtained by a pairwise t-test with Bonferroni correction. Significant differences are shown in bold.

|  | **n1** | **n2** | **p** | **p.adj** |
| --- | --- | --- | --- | --- |
| ***All specimens*** |  |  |  |  |
| **C. buettikoferi - C. cf. grandiceps** | 107 | 15 | 0.000 | **0.006** |
| **C. buettikoferi - C. foxi** | 107 | 24 | 0.000 | **0.000** |
| **C. buettikoferi - C. grandiceps** | 107 | 25 | 0.000 | **0.000** |
| C. buettikoferi - C. longipes | 107 | 9 | 0.039 | 0.818 |
| C. buettikoferi - C. poensis | 107 | 174 | 0.009 | 0.182 |
| **C. buettikoferi - C. theresae** | 107 | 74 | 0.000 | **0.000** |
| **C. cf. grandiceps - C. foxi** | 15 | 24 | 0.000 | **0.000** |
| **C. cf. grandiceps - C. grandiceps** | 15 | 25 | 0.000 | **0.000** |
| C. cf. grandiceps - C. longipes | 15 | 9 | 0.496 | 1.000 |
| C. cf. grandiceps - C. poensis | 15 | 174 | 0.012 | 0.244 |
| C. cf. grandiceps - C. theresae | 15 | 74 | 0.062 | 1.000 |
| **C. foxi - C. grandiceps** | 24 | 25 | 0.000 | **0.000** |
| **C. foxi - C. longipes** | 24 | 9 | 0.000 | **0.000** |
| **C. foxi - C. poensis** | 24 | 174 | 0.000 | **0.000** |
| **C. foxi - C. theresae** | 24 | 74 | 0.000 | **0.000** |
| **C. grandiceps - C. longipes** | 25 | 9 | 0.000 | **0.000** |
| **C. grandiceps - C. poensis** | 25 | 174 | 0.000 | **0.000** |
| **C. grandiceps - C. theresae** | 25 | 74 | 0.000 | **0.000** |
| C. longipes - C. poensis | 9 | 174 | 0.249 | 1.000 |
| C. longipes - C. theresae | 9 | 74 | 0.021 | 0.442 |
| **C. poensis - C. theresae** | 174 | 74 | 0.000 | **0.000** |
| ***Adults*** |  |  |  |  |
| **C. buettikoferi - C. cf. grandiceps** | 65 | 8 | 0.000 | **0.010** |
| **C. buettikoferi - C. foxi** | 65 | 14 | 0.000 | **0.006** |
| **C. buettikoferi - C. grandiceps** | 65 | 10 | 0.000 | **0.000** |
| **C. buettikoferi - C. longipes** | 65 | 9 | 0.001 | **0.023** |
| **C. buettikoferi - C. poensis** | 65 | 75 | 0.001 | **0.016** |
| **C. buettikoferi - C. theresae** | 65 | 48 | 0.000 | **0.000** |
| **C. cf. grandiceps - C. foxi** | 8 | 14 | 0.000 | **0.000** |
| **C. cf. grandiceps - C. grandiceps** | 8 | 10 | 0.000 | **0.000** |
| C. cf. grandiceps - C. longipes | 8 | 9 | 0.752 | 1.000 |
| C. cf. grandiceps - C. poensis | 8 | 75 | 0.045 | 0.950 |
| C. cf. grandiceps - C. theresae | 8 | 48 | 0.278 | 1.000 |
| **C. foxi - C. grandiceps** | 14 | 10 | 0.000 | **0.000** |
| **C. foxi - C. longipes** | 14 | 9 | 0.000 | **0.000** |
| **C. foxi - C. poensis** | 14 | 75 | 0.000 | **0.000** |
| **C. foxi - C. theresae** | 14 | 48 | 0.000 | **0.000** |
| **C. grandiceps - C. longipes** | 10 | 9 | 0.000 | **0.000** |
| **C. grandiceps - C. poensis** | 10 | 75 | 0.000 | **0.000** |
| **C. grandiceps - C. theresae** | 10 | 48 | 0.000 | **0.000** |
| C. longipes - C. poensis | 9 | 75 | 0.093 | 1.000 |
| C. longipes - C. theresae | 9 | 48 | 0.119 | 1.000 |
| **C. poensis - C. theresae** | 75 | 48 | 0.000 | **0.000** |

Table 4: Shape comparisons between species for all specimens and adults only. Values were obtained using ProcD.lm and pairwise, by comparing distance between vectors. Significant differences are shown in bold.

|  | **d** | **UCL (95%)** | **Z** | **Pr > d** |
| --- | --- | --- | --- | --- |
| ***All specimens*** |  |  |  |  |
| ***C. buettikoferi - C. cf. grandiceps*** | 0.018 | 0.009 | 3.788 | **0.001** |
| ***C. buettikoferi - C. foxi*** | 0.012 | 0.008 | 3.275 | **0.002** |
| ***C. buettikoferi - C. grandiceps*** | 0.025 | 0.007 | 6.765 | **0.001** |
| ***C. buettikoferi - C. longipes*** | 0.031 | 0.011 | 5.821 | **0.001** |
| ***C. buettikoferi - C. poensis*** | 0.009 | 0.004 | 4.140 | **0.001** |
| ***C. buettikoferi - C. theresae*** | 0.016 | 0.005 | 5.805 | **0.001** |
| ***C. cf. grandiceps - C. foxi*** | 0.022 | 0.011 | 4.042 | **0.001** |
| ***C. cf. grandiceps - C. grandiceps*** | 0.035 | 0.011 | 5.741 | **0.001** |
| ***C. cf. grandiceps - C. longipes*** | 0.017 | 0.015 | 2.315 | **0.007** |
| ***C. cf. grandiceps - C. poensis*** | 0.021 | 0.009 | 4.345 | **0.001** |
| *C. cf. grandiceps - C. theresae* | 0.008 | 0.010 | 1.035 | 0.148 |
| ***C. foxi - C. grandiceps*** | 0.017 | 0.010 | 3.578 | **0.001** |
| ***C. foxi - C. longipes*** | 0.029 | 0.012 | 5.420 | **0.001** |
| ***C. foxi - C. poensis*** | 0.011 | 0.007 | 3.209 | **0.001** |
| ***C. foxi - C. theresae*** | 0.021 | 0.008 | 4.711 | **0.001** |
| ***C. grandiceps - C. longipes*** | 0.041 | 0.013 | 6.347 | **0.001** |
| ***C. grandiceps - C. poensis*** | 0.022 | 0.007 | 6.041 | **0.001** |
| ***C. grandiceps - C. theresae*** | 0.035 | 0.008 | 8.369 | **0.001** |
| ***C. longipes - C. poensis*** | 0.032 | 0.011 | 6.041 | **0.001** |
| ***C. longipes - C. theresae*** | 0.019 | 0.011 | 3.714 | **0.001** |
| ***C. poensis - C. theresae*** | 0.018 | 0.005 | 6.388 | **0.001** |
| ***Adults*** |  |  |  |  |
| ***C. buettikoferi - C. cf. grandiceps*** | 0.016 | 0.012 | 2.613 | **0.008** |
| ***C. buettikoferi - C. foxi*** | 0.011 | 0.010 | 2.288 | **0.013** |
| ***C. buettikoferi - C. grandiceps*** | 0.024 | 0.011 | 4.771 | **0.001** |
| ***C. buettikoferi - C. longipes*** | 0.027 | 0.011 | 4.873 | **0.001** |
| ***C. buettikoferi - C. poensis*** | 0.009 | 0.006 | 3.471 | **0.001** |
| ***C. buettikoferi - C. theresae*** | 0.017 | 0.006 | 5.304 | **0.001** |
| ***C. cf. grandiceps - C. foxi*** | 0.017 | 0.015 | 2.048 | **0.017** |
| ***C. cf. grandiceps - C. grandiceps*** | 0.030 | 0.015 | 4.505 | **0.001** |
| *C. cf. grandiceps - C. longipes* | 0.013 | 0.015 | 0.909 | 0.190 |
| ***C. cf. grandiceps - C. poensis*** | 0.019 | 0.012 | 3.336 | **0.001** |
| *C. cf. grandiceps - C. theresae* | 0.008 | 0.012 | 0.351 | 0.371 |
| ***C. foxi - C. grandiceps*** | 0.019 | 0.013 | 3.022 | **0.001** |
| ***C. foxi - C. longipes*** | 0.024 | 0.014 | 3.486 | **0.001** |
| ***C. foxi - C. poensis*** | 0.010 | 0.010 | 1.659 | **0.051** |
| ***C. foxi - C. theresae*** | 0.018 | 0.010 | 3.557 | **0.001** |
| ***C. grandiceps - C. longipes*** | 0.035 | 0.015 | 5.406 | **0.001** |
| ***C. grandiceps - C. poensis*** | 0.020 | 0.011 | 4.352 | **0.001** |
| ***C. grandiceps - C. theresae*** | 0.032 | 0.011 | 6.154 | **0.001** |
| ***C. longipes - C. poensis*** | 0.028 | 0.011 | 4.715 | **0.001** |
| ***C. longipes - C. theresae*** | 0.016 | 0.012 | 2.683 | **0.006** |
| ***C. poensis - C. theresae*** | 0.018 | 0.006 | 6.086 | **0.001** |

Table 5: Allometric slope comparisons for all specimens for species for which N ≥ 9. Significant differences are shown in bold.

|  | **r** | **angle** | **UCL (95%)** | **Z** | **Pr > angle** |
| --- | --- | --- | --- | --- | --- |
| *C. buettikoferi - C. cf. grandiceps* | 0.43 | 64.83 | 88.16 | 0.60 | 0.277 |
| *C. buettikoferi - C. foxi* | 0.85 | 32.14 | 80.00 | -1.44 | 0.923 |
| ***C. buettikoferi - C. grandiceps*** | **0.42** | **65.00** | **63.52** | **1.77** | **0.041** |
| *C. buettikoferi - C. longipes* | 0.64 | 49.85 | 83.22 | -0.12 | 0.550 |
| ***C. buettikoferi - C. poensis*** | **0.61** | **52.66** | **37.18** | **2.91** | **0.002** |
| *C. buettikoferi - C. theresae* | 0.80 | 37.09 | 51.77 | 0.48 | 0.311 |
| *C. cf. grandiceps - C. foxi* | 0.40 | 66.13 | 98.68 | 0.08 | 0.467 |
| *C. cf. grandiceps - C. grandiceps* | 0.56 | 55.99 | 95.25 | -0.30 | 0.613 |
| *C. cf. grandiceps - C. longipes* | 0.47 | 62.26 | 102.19 | -0.23 | 0.590 |
| *C. cf. grandiceps - C. poensis* | 0.56 | 56.13 | 87.32 | 0.20 | 0.422 |
| *C. cf. grandiceps - C. theresae* | 0.44 | 64.14 | 90.16 | 0.46 | 0.322 |
| *C. foxi - C. grandiceps* | 0.50 | 60.30 | 88.59 | 0.15 | 0.442 |
| *C. foxi - C. longipes* | 0.63 | 50.61 | 98.81 | -0.86 | 0.804 |
| *C. foxi - C. poensis* | 0.60 | 53.14 | 82.51 | 0.30 | 0.397 |
| *C. foxi - C. theresae* | 0.90 | 25.56 | 86.49 | -2.50 | 0.994 |
| *C. grandiceps - C. longipes* | 0.24 | 75.94 | 92.99 | 0.96 | 0.166 |
| *C. grandiceps - C. poensis* | 0.82 | 34.54 | 62.21 | -0.45 | 0.679 |
| *C. grandiceps - C. theresae* | 0.51 | 59.62 | 70.16 | 1.06 | 0.154 |
| *C. longipes - C. poensis* | 0.37 | 68.55 | 83.34 | 1.04 | 0.153 |
| *C. longipes - C. theresae* | 0.57 | 55.12 | 86.18 | 0.07 | 0.471 |
| ***C. poensis - C. theresae*** | **0.53** | **58.00** | **50.78** | **2.12** | **0.015** |

Table 6: Allometric vector length comparisons for all specimens for species for which N ≥ 9. Significant differences are shown in bold.

|  | **d** | **UCL (95%)** | **Z** | **Pr > d** |
| --- | --- | --- | --- | --- |
| *C. buettikoferi - C. cf. grandiceps* | 0.13 | 0.18 | 0.82 | 0.214 |
| ***C. buettikoferi - C. foxi*** | **0.17** | **0.14** | **1.99** | **0.02** |
| ***C. buettikoferi - C. grandiceps*** | **0.12** | **0.10** | **1.95** | **0.018** |
| *C. buettikoferi - C. longipes* | 0.03 | 0.15 | -0.68 | 0.741 |
| *C. buettikoferi - C. poensis* | 0.04 | 0.05 | 1.39 | 0.083 |
| ***C. buettikoferi - C. theresae*** | **0.15** | **0.06** | **3.36** | **0.001** |
| *C. cf. grandiceps - C. foxi* | 0.04 | 0.15 | -0.15 | 0.569 |
| *C. cf. grandiceps - C. grandiceps* | 0.01 | 0.17 | -1.30 | 0.885 |
| *C. cf. grandiceps - C. longipes* | 0.10 | 0.15 | 0.88 | 0.194 |
| *C. cf. grandiceps - C. poensis* | 0.09 | 0.19 | 0.15 | 0.459 |
| *C. cf. grandiceps - C. theresae* | 0.02 | 0.18 | -1.01 | 0.826 |
| *C. foxi - C. grandiceps* | 0.05 | 0.13 | 0.38 | 0.375 |
| ***C. foxi - C. longipes*** | **0.14** | **0.13** | **1.71** | **0.038** |
| *C. foxi - C. poensis* | 0.13 | 0.14 | 1.39 | 0.067 |
| *C. foxi - C. theresae* | 0.02 | 0.14 | -0.95 | 0.816 |
| *C. grandiceps - C. longipes* | 0.08 | 0.14 | 0.81 | 0.231 |
| *C. grandiceps - C. poensis* | 0.08 | 0.09 | 1.23 | 0.115 |
| *C. grandiceps - C. theresae* | 0.04 | 0.09 | 0.19 | 0.457 |
| *C. longipes - C. poensis* | 0.01 | 0.16 | -1.53 | 0.927 |
| *C. longipes - C. theresae* | 0.12 | 0.15 | 1.17 | 0.126 |
| ***C. poensis - C. theresae*** | **0.11** | **0.06** | **2.79** | **0.002** |

Table 7: Multivariate regressions of size on skull shape PCs for individual species datasets. Significant results are shown in bold.

|  | Df | SS | MS | R^2^ | F | Z | P value |
| --- | --- | --- | --- | --- | --- | --- | --- |
| ***C. poensis*** | 1 | 0.004 | 0.004 | **0.061** | 11.118 | 4.689 | **0.001** |
| ***C. buettikoferi*** | 1 | 0.002 | 0.002 | **0.038** | 4.164 | 2.862 | **0.003** |
| ***C. foxi*** | 1 | 0.001 | 0.001 | **0.120** | 2.999 | 2.175 | **0.013** |
| ***C. theresae*** | 1 | 0.005 | 0.005 | **0.194** | 17.349 | 5.438 | **0.001** |
| ***C. longipes*** | 1 | 0.000 | 0.000 | 0.115 | 0.908 | -0.003 | 0.487 |
| ***C. grandiceps*** | 1 | 0.001 | 0.001 | **0.124** | 3.256 | 2.131 | **0.020** |
| ***C. cf. grandiceps*** | 1 | 0.000 | 0.000 | 0.056 | 0.773 | -0.306 | 0.626 |

1. Schneider, C. A., Rasband, W. S. & Eliceiri, K. W. NIH Image to ImageJ: 25 years of image analysis. *Nat. Methods* **9**, 671–675 (2012).

2. R Core Team (2021). *R: A language and environment for statistical computing.* (R Foundation for Statistical Computing, Vienna, Austria.).

3. Bonhomme, V., Picq, S., Gaucherel, C. & Claude, J. Momocs: Outline Analysis Using R. *J. Stat. Softw.* **56**, 1–24 (2014).

4. Bookstein, F. L. *Morphometric Tools for Landmark Data: Geometry and Biology*. (Cambridge University Press, 1991). doi:10.1017/CBO9780511573064.

5. Rohlf, F. J. & Slice, D. Extensions of the Procrustes Method for the Optimal Superimposition of Landmarks. *Syst. Zool.* **39**, 40–59 (1990).

6. Adams, D. C. & Otárola-Castillo, E. geomorph: an r package for the collection and analysis of geometric morphometric shape data. *Methods Ecol. Evol.* **4**, 393–399 (2013).

7. Gunz, P. & Mitteroecker, P. Semilandmarks: a method for quantifying curves and surfaces. *Hystrix Ital. J. Mammal.* **24**, 103–109 (2013).

8. Parsons, P. A. Fluctuating asymmetry: a biological monitor of environmental and genomic stress. *Heredity* **68**, 361–364 (1992).

9. Baylac, M. & Frieß, M. Fourier Descriptors, Procrustes Superimposition, and Data Dimensionality: An Example of Cranial Shape Analysis in Modern Human Populations. in *Modern Morphometrics in Physical Anthropology* (ed. Slice, D. E.) 145–165 (Springer US, 2005). doi:10.1007/0-387-27614-9_6.

10. Nicolas, V. *et al.* Multilocus phylogeny of the Crocidura poensis species complex (Mammalia, Eulipotyphla): Influences of the palaeoclimate on its diversification and evolution. *J. Biogeogr.* **46**, 871–883 (2019).

11. Konečný, A., Hutterer, R., Meheretu, Y. & Bryja, J. Two new species of Crocidura (Mammalia: Soricidae) from Ethiopia and updates on the Ethiopian shrew fauna. *J. Vertebr. Biol.* **69**, 20064.1 (2020).

12. Lanfear, R., Frandsen, P. B., Wright, A. M., Senfeld, T. & Calcott, B. PartitionFinder 2: New Methods for Selecting Partitioned Models of Evolution for Molecular and Morphological Phylogenetic Analyses. *Mol. Biol. Evol.* **34**, 772–773 (2017).

13. Drummond, A. J., Suchard, M. A., Xie, D. & Rambaut, A. Bayesian Phylogenetics with BEAUti and the BEAST 1.7. *Mol. Biol. Evol.* **29**, 1969–1973 (2012).

14. Ferreira, M. A. R. & Suchard, M. A. Bayesian analysis of elapsed times in continuous-time Markov chains. *Can. J. Stat.* **36**, 355–368 (2008).

15. Rambaut, A., Drummond, A. J., Xie, D., Baele, G. & Suchard, M. A. Posterior Summarization in Bayesian Phylogenetics Using Tracer 1.7. *Syst. Biol.* **67**, 901–904 (2018).

16. Drummond, A. J. & Rambaut, A. BEAST: Bayesian evolutionary analysis by sampling trees. *BMC Evol. Biol.* **7**, 214 (2007).

17. Dubey, S. *et al.* Biogeographic origin and radiation of the Old World crocidurine shrews (Mammalia: Soricidae) inferred from mitochondrial and nuclear genes. *Mol. Phylogenet. Evol.* **48**, 953–963 (2008).
